# Radiation-Induced DNA Damage in Uveal Melanoma Is Influenced by Dose Delivery and Chromosome 3 Status

**DOI:** 10.1167/iovs.65.6.7

**Published:** 2024-06-04

**Authors:** Aysegül Tura, Yingda Zhu, Siranush Vardanyan, Michelle Prasuhn, Vinodh Kakkassery, Julia Lüke, Hartmut Merz, Frank Paulsen, Dirk Rades, Florian Cremers, Karl-Ulrich Bartz-Schmidt, Salvatore Grisanti

**Affiliations:** 1 Department of Ophthalmology, University Clinic Schleswig-Holstein (UKSH), University of Lübeck, Lübeck, Germany; 2 Department of Ophthalmology, The First Affiliated Hospital of Xiamen University, School of Medicine, Xiamen University, Xiamen, China; 3 Reference Center for Lymph Node Pathology and Hematopathology, Lübeck, Germany; 4 Department of Radiation Oncology, Eberhard Karls University Tübingen, Tübingen, Germany; 5 Department of Radiation Oncology, University Clinic Schleswig-Holstein (UKSH), University of Lübeck, Lübeck, Germany; 6 Department of Ophthalmology, Eberhard Karls University Tübingen, Tübingen, Germany

**Keywords:** uveal melanoma, stereotactic radiotherapy (SRT), monosomy 3, DNA break

## Abstract

**Purpose:**

The purpose of this study was to analyze the extent of DNA breaks in primary uveal melanoma (UM) with regard to radiotherapy dose delivery (single-dose versus fractionated) and monosomy 3 status.

**Methods:**

A total of 54 patients with UM were included. Stereotactic radiotherapy (SRT) was performed in 23 patients, with 8 undergoing single-dose SRT (sdSRT) treatment and 15 receiving fractionated SRT (fSRT). DNA breaks in the enucleated or endoresected tumors were visualized by a TUNEL assay and quantified by measuring the TUNEL-positive area. Protein expression was analyzed by immunohistochemistry. Co-detection of chromosome 3 with proteins was performed by immuno-fluorescent in situ hybridization.

**Results:**

The amount of DNA breaks in the total irradiated group was increased by 2.7-fold (*P* < 0.001) compared to non-irradiated tissue. Tumors treated with fSRT were affected more severely, showing 2.1-fold more DNA damage (*P* = 0.007) compared to the cases after single (high) dose irradiation (sdSRT). Monosomy 3 tumors showed less DNA breaks compared to disomy 3 samples (*P* = 0.004). The presence of metastases after radiotherapy correlated with monosomy 3 and less DNA breaks compared to patients with non-metastatic cancer in the combined group with fSRT and sdSRT (*P* < 0.05).

**Conclusions:**

Fractionated irradiation led to more DNA damage than single-dose treatment in primary UM. As tumors with monosomy 3 showed less DNA breaks than those with disomy 3, this may indicate that they are less radiosensitive, which may influence the efficacy of irradiation.

Uveal melanoma (UM) is the most frequently encountered primary intraocular malignancy in adults.^1^^–^^3^ Due to the absence of clinically evident metastases in the majority of patients at initial presentation, the management of this malignancy mainly focuses on the primary tumor.^2^^,^^4^ Radiation therapy is the predominant approach for the eye-preserving treatment.^3^ Dependent on the tumor size and location, irradiation can be administered either as brachytherapy or by an external beam with charged-particles or photons.^2^^–^^5^ Although the physical properties of the proton beam appear more attractive, recent in vitro and clinical head-to-head studies showed no difference in tumor control when protons or photons were applied.^6^^,^^7^ In stereotactic radiotherapy (SRT), the radiation is delivered either as a single dose SRT (sdSRT) at high dose (e.g. 25 Gray [Gy])^8^ or as fractionated SRT (fSRT), consisting of several lower dose fractions (e.g. 5 × 10 Gy or 6 × 8 Gy).^2^^,^^4^^,^^7^ Radiotherapy, however, is a two-edged sword: the primary goal is to destroy the tumor. Indeed, insufficient tumor control and local recurrence are associated with an increased risk of fatal metastases.^9^ The second goal is to avoid or reduce severe side effects caused by irradiation, as ocular complications may induce vision loss or necessitate a secondary enucleation.^10^

An earlier study has reported variations in the intrinsic radiosensitivity of UM cell lines, suggesting that irradiation with 5 × 8 Gy would be sufficient to destroy a radioresistant tumor with a volume of 1 mm^3^ (with approximately 10^9^ cells), whereas this treatment would be excessive in the case of more sensitive UM cells.^11^ The variance in radiosensitivity of the UM cell lines presumably arises due to the differences in the capacity to repair DNA damage.^12^ A reduction in DNA content, which serves as an indicator of sublethal cell damage, was indeed observed in primary UMs within the first 6 months after fSRT despite the vital appearance of the majority of the tumor cells.^13^ However, it remains unknown, which tumor markers can distinguish the UM patients at higher risk of radioresistance.

The most important risk factor for the development of UM metastasis is the loss of one copy of chromosome 3 (monosomy 3) in the primary tumor cells.^5^^,^^14^ Tumor size correlates with monosomy 3, with larger primary tumors showing a higher proportion of cells having monosomy 3, possibly due to the longer growth period which would increase the likelihood of this anomaly. Accordingly, the larger UMs are associated with a worse prognosis.^15^ However, also smaller UMs with monosomy 3, treated with brachytherapy, have shown a worse prognosis compared to disomy 3 tumors.^15^ Nevertheless, conflicting findings exist regarding the influence of the chromosome 3 status on the efficacy of the radiotherapy, with no significant correlation between the monosomy 3 and regression rate, as measured by tumor height before and after brachytherapy.^16^

Interestingly, the differential expression of several genes that regulate DNA repair was observed in UM samples with regard to the development of metastases and presence of unfavorable prognostic factors such as monosomy 3.^17^ However, we found no reports that investigated the relation of DNA damage with chromosome 3 status and the dose delivery regimen used by SRT.

To elucidate these aspects, we determined the presence of DNA breaks in 54 primary UMs with or without SRT, using a histopathological analysis of DNA breaks. The earliest group of cases had been treated with SRT by applying a single radiation dose. Although high single doses may induce severe side effects,^10^ this treatment was based on an earlier study which reported a more profound cell damage in choroidal melanoma cells that received a single high dose as opposed to the fractioned irradiation.^18^ Based on other experimental and clinical studies,^12^^,^^19^ however, a fractionated irradiation was chosen for the second cohort (fSRT). The control tissue originates from patients who underwent planned enucleation without previous treatment during the same period. The DNA breaks were visualized by the TUNEL assay and evaluated with regard to the clinical and histopathological factors, including chromosome 3 status and development of metastatic disease.

## Methods

### Patient Characteristics and Tumor Samples

This study was conducted retrospectively on a total of 54 eyes containing a primary tumor, of which 23 had previously been irradiated in 2 clinics. A total of 29 patients was treated at the University Eye Clinic in Tübingen, Germany (tumor diagnosis between 1982 and 2008), with 8 patients receiving single-dose SRT and 21 a primary enucleation. A total of 25 patients was treated at the University Eye Clinic in Lübeck, Germany (tumor diagnosis between December 2009 and November 2015), with 15 patients treated with fractionated doses (fSRT) and 10 undergoing primary enucleation. All patients treated with single-dose irradiation (sdSRT) had undergone enucleation. The reason for enucleation of those patients could not be ascertained due to the limited clinical data. Endoresection was offered to the patients in the fSRT group to reduce the radiation-related complications, such as progressive retinal exudation or tumor necrosis.^19^ Planned endoresection following primary radiotherapy was performed as neoadjuvant therapy to minimize the risk of viable tumor cell dissemination during surgery.^20^ For endoresection, patients were selected based on the tumor location and size. Within the fSRT group, 9 of the 15 tumors (60%) were resected within 3 months following radiotherapy. The remaining patients receiving fSRT (6 of 15) had been monitored longer (for up to 16 months) and tumor resection had been performed based on the development of radiation-induced complications. Four eyes treated with fractionated irradiation were enucleated because of irradiation-related complications or suspected growth.

The analysis of human tissue samples was approved by the local ethics committees of the University Hospital Tübingen (reference: 90/2003V, date: April 9, 2003) and the University of Lübeck (reference: 10-200, date: December 17, 2010). The study was conducted in accordance with the guidelines of the Declaration of Helsinki of 1975, revised in 2013. All patients gave informed consent prior to their inclusion into the study.

The diagnosis of UM was ascertained by specialized ophthalmologists via clinical examinations and by a certified pathologist (author H.M.) based on the histopathological analysis of the obtained tissue. Tumor size, exact intraocular localization, and ciliary body involvement were determined by standardized A and B scans and ultrasound biomicroscopy (Dicon Ultrasound Biomicroscope Plus, Model P45, UBM Plus; Paradigm Medical Industries Inc., Salt Lake City, UT, USA [Tübingen cohort] or VuMax II, Sonomed Inc., New Hyde Park, NY, USA [Lübeck cohort]). The development of systemic metastases was monitored by liver function tests (alkaline phosphatase, aspartate aminotransferase, alanine aminotransferase, and bilirubin), ultrasound of the abdomen, and computer tomography of the chest and abdomen.

The endoresection samples were intact or dissociated tissue pieces. All samples were fixed in formalin, embedded in paraffin, processed as 6-µm paraffin sections, and analyzed anonymously. The clinical data of the patients and the histopathological characteristics of their primary tumors are listed in Tables 1 and 2.

**Table 1.** Clinical Data and Histopathological Features

	**All** *n* = 54 (100%)	**no RT** *n* = 31 (57.4%)	**SRT** *n* = 23 (42.6%)	***P*** SRT vs. no RT	**sdSRT** *n* = 8 (14.8%)	***P*** sdSRT vs. no RT	**fSRT** *n* = 15 (27.8%)	***P*** fSRT vs. no RT	***P*** sdSRT vs. fSRT
Age, y									
Mean ± SD	65.5 ± 13.4	65.5 ± 16.1	65.4 ± 9.0	0.63	69.6 ± 7.1	0.81	63.1 ± 9.3	0.41	0.10
Median	66.5	72.0	65.0		68.5		62.0		
Min-Max	37–90	37–90	51–83		61–82		51–83		
Sex									
F, *n* (%)	24 (44.4)	17 (31.5)	7 (13.0)	0.10	1 (1.9)	**0.049**	6 (11.1)	0.53	0.35
M, *n* (%)	30 (55.6)	14 (25.9)	16 (29.6)		7 (13.0)		9 (16.7)		
Largest basal diameter, mm^*^									
Mean ± SD	15.4 ± 5.2	16.4 ± 5.6	14.0 ± 4.4	**0.048**	14.9 ± 5.6	0.41	13.6 ± 3.8	**0.041**	0.86
Median	16.0	17.0	14.2		14.0		14.4		
Min	1.4	1.4	6.0		8.0		6.0		
Max	28.0	28.0	23.0		23.0		18.3		
Tumor thickness, mm ^†^									
Mean ± SD	7.9 ± 4.3	9.2 ± 4.8	6.2 ± 2.9	**0.017**	7.1 ± 3.2	0.27	5.8 ± 2.8	**0.016**	0.55
Median	8.0	9.0	6.5		8.0		6.4		
Min	0.5	0.9	0.5		3.0		0.5		
Max	22.0	22.0	12.0		12.0		8.7		
Ciliary body invasion									
No, *n* (%)	35 (64.8)	15 (27.8)	20 (37.0)	**0.004**	5 (9.3)	0.70	15 (27.8)	**0.001**	**0.032**
Yes, *n* (%)	19 (35.2)	16 (29.6)	3 (5.6)		3 (5.6)		0 (0.0)		
Optic nerve invasion									
No, *n* (%)	50 (92.6)	30 (55.6)	20 (37.0)	0.30	8 (14.8)	1.00	12 (22.2)	0.10	0.53
Yes, *n* (%)	4 (7.4)	1 (1.9)	3 (5.6)		0 (0.0)		3 (5.6)		
Tumor cell morphology									
Spindle/mixed, *n* (%)	40 (74.1)	22 (40.7)	18 (33.3)	0.76	6 (11.1)	1.00	12 (22.2)	0.72	1.00
Epithelioid, *n* (%)	14 (25.9)	9 (16.7)	5 (9.3)		2 (3.7)		3 (5.6)		
Mitotic figures ^‡^									
Mean ± SD	2.5 ± 1.9	3.1 ± 1.9	1.6 ± 1.6	**0.008**	2.5 ± 2	0.47	0.9 ± 0.9	**0.001**	0.10
Median	2	3	1		3		1		
Min	0	0	0		0		0		
Max	6	6	5		5		3		
Necrosis									
0–10%, *n* (%)	41 (75.9)	24 (44.4)	17 (31.5)	1.00	7 (13.0)	1.00	10 (18.5)	0.49	0.37
>10%–33%, *n* (%)	13 (24.1)	7 (13.0)	6 (11.1)		1 (1.9)		5 (9.3)		
Tumor vascularization (vWF+ area, %)									
Mean ± SD	2.1 ± 1.7	2.2 ± 2.1	2.0 ± 1.0	0.58	2.0 ± 1.2	0.68	1.9 ± 0.9	0.65	1.00
Median	1.6	1.5	1.9		1.9		1.7		
Min	0.7	0.7	0.7		0.8		0.7		
Max	10.5	10.5	4.5		4.5		3.7		
Macrophage infiltration (CD68+ cells/mm^2^)									
Mean ± SD	17.0 ± 15.7	17.2 ± 18.4	16.7 ± 11.4	0.50	19.6 ± 15.6	0.47	15.2 ± 8.7	0.68	0.70
Median	12.6	11.9	17.5		13.7		17.8		
Min	1.3	1.3	2.6		4.7		2.6		
Max	82.5	82.5	51.6		51.6		33.0		
Follow-up, mo									
Mean ± SD	58.9 ± 42.2	52.5 ± 40.5	68.5 ± 43.3	0.11	76.5 ± 55.2	0.28	64.3 ± 37.0	0.16	0.68
Median	51.5	48.0	64.0		72.0		64.0		
Min-Max	10–144	10–144	12–144		12–144		13–129		
Metastatic disease									
No, *n* (%)	24 (44.4)	12 (22.2)	12 (22.2)	0.41	4 (7.4)	0.69	8 (14.8)	0.53	1.00
Yes, *n* (%)	30 (55.6)	19 (35.2)	11 (20.4)		4 (7.4)		7 (13.0)		

fSRT, fractionated stereotactic radiotherapy; Max, maximum; Min, minimum; mm, millimeter; n, number; RT, radiotherapy; SD, standard deviation; sdSRT, single-dose stereotactic radiotherapy; SRT, stereotactic radiotherapy.

The *P* values were determined by the Mann-Whitney *U* test with normal approximation (applying continuity correction in case of ties) for numerical variables and by Fisher's Exact test for the categorical parameters.

* Data missing from one patient.

† Data missing from three patients.

‡ Data missing from four patients.

**Table 2.** DNA Damage and Chromosome 3 Status

	**All** *n* = 54 (100%)	**no RT** *n* = 31 (57.4%)	**SRT** *n* = 23 (42.6%)	***P*** SRT vs. no RT	**sdSRT** *n* = 8 (14.8%)	***P*** sdSRT vs. no RT	**fSRT** *n* = 15 (27.8%)	***P*** fSRT vs. no RT	***P*** sdSRT vs. fSRT
DNA damage (TUNEL-positive) area (%)									
Mean ± SD	8.4 ± 6.9	4.9 ± 4.0	13.1 ± 7.3	**<0.001**	7.6 ± 5.5	0.21	15.9 ± 6.6	**<0.001**	**0.007**
Median	6.5	4.0	14.0		7.3		15.1		
Min	0.0	0.0	1.1		1.1		3.4		
Max	26.3	13.9	26.3		15.0		26.3		
Chromosome 3 status									
Disomy 3, *n* (%)	27 (50.0)	15 (27.8)	12 (22.2)	1.00	3 (5.6)	0.70	9 (16.7)	0.54	0.40
Monosomy 3, *n* (%)	27 (50.0)	16 (29.6)	11 (20.4)		5 (9.3)		6 (11.1)		

fSRT, fractionated stereotactic radiotherapy; Max, maximum; Min, minimum; *n*, number; RT, radiotherapy; SD, standard deviation; sdSRT, single-dose stereotactic radiotherapy; SRT, stereotactic radiotherapy.

The *P* values were determined by the Mann-Whitney *U* test with normal approximation for numerical variables and by Fisher's Exact test for the categorical parameters.

### Stereotactic Radiotherapy

SRT was performed (*n* = 23 of the 54 patients, 43%) with photons generated by a 6-megavolt linear accelerator.^8^^,^^21^^–^^23^ Single-dose irradiation (sdSRT) was initially performed using retrobulbar anesthesia to immobilize the eye, which was later substituted by a functional eye immobilization device for active fixation by the patient.^23^ Similarly, fractionated irradiation was performed using a modified mask that enabled the controlled fixation of a light point. During treatments, the position of the pupil was monitored with an infrared camera. The treatment planning was aided by computed tomography and magnetic resonance imaging to obtain the density information for dose calculation and to demarcate the target from the surrounding tissues, respectively. Single dose SRT (sdSRT) was performed with an aimed dose of 25 Gy to the tumor margin and a surrounding isodose between 50% and 90%. Fractionated doses (fSRT) were given in 5 to 8 fractions, reaching a prescribed (total) dose of 40 to 60 Gy with an encompassing 80%-isodose and a near-minimum dose (D98%) of 39 to 54 Gy. The time interval between irradiation and surgery was consistently 2 weeks in the single-dose (sdSRT) group (*n* = 8), but ranged between 1 and 16 months in the fSRT group (*n* = 15).

### TUNEL Assay

After deparaffinization in xylene, the sections of tumor samples were rehydrated in a descending series of ethanol (99%–50%) followed by triple distilled water and phosphate-buffered saline (PBS) for 5 minutes each. DNA breaks were detected by a chromogenic TUNEL assay following the manufacturer's instructions (Merck, Darmstadt, Germany, catalog number: S7100). Briefly, the sections were treated with 20 µg/mL Proteinase K in PBS for 15 minutes at room temperature in a humid chamber, washed twice for 2 minutes each in triple-distilled water, blocked in 3% hydrogen peroxide in PBS for 5 minutes, washed with PBS, covered with equilibration buffer for 10 seconds, and incubated with the terminal deoxynucleotidyl transferase enzyme (75 µL/slide) at 37°C for 1 hour. The negative controls were kept in the reaction buffer alone. Subsequently, the sections were shaken in the Stop/Wash Buffer for 15 seconds, incubated for 10 minutes at room temperature, and washed 3 times for 1 minute in PBS. Sections were then incubated with the anti-digoxigenin conjugate for 30 minutes at room temperature, washed with PBS, treated with the horseradish peroxidase (HRP) Green substrate (42 Is, Bremerhaven, Germany, 60 µL HRP Green chromogen in 1 mL HRP Green buffer) for 10 minutes, and rinsed with triple-distilled water. Counterstaining of the nuclei was performed with nuclear fast red for 10 minutes. After a final rinse in triple-distilled water for 1 minute, the sections were immersed in 2 cuvettes of 100% N-butanol for 10 times each, followed by a third cuvette of 100% N-butanol for 30 seconds and 3 changes of xylene for 2 minutes each. The sections were covered with Entellan (Merck) and sealed with a coverglass.

### Analysis of TUNEL-Positive Areas

**Figure 1.**
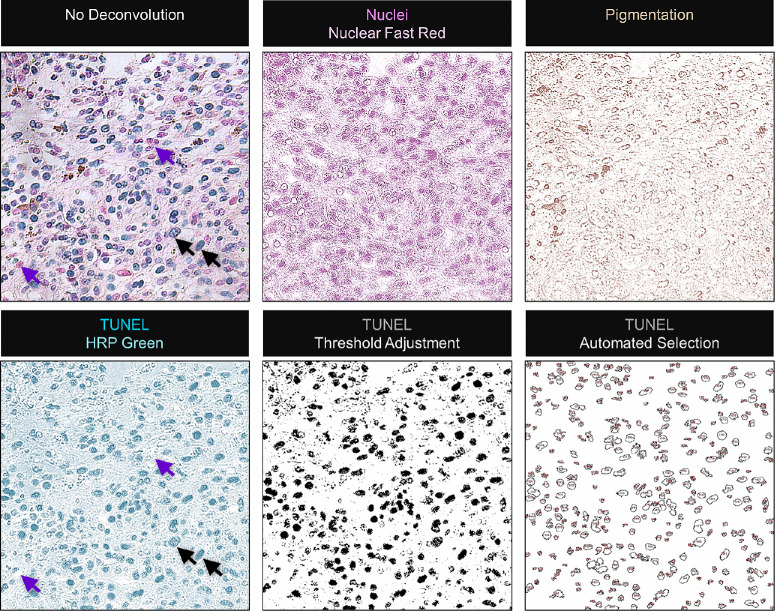
Automated selection of the TUNEL-positive areas on the light microscopy images. The TUNEL reaction was visualized by using the horseradish peroxidase (HRP) green chromogen, whereas the nuclei were counterstained with nuclear fast red. Color deconvolution with user-defined settings was performed to separate the TUNEL signals (*blue-green*) from the nuclear staining and brown pigmentation with minimal overlap. The TUNEL panel was then processed for threshold adjustment for the automated selection of the positive regions. The sum of the TUNEL-positive areas was calculated and presented as the percentage of the total area that was analyzed in each tumor. Images were acquired at a magnification of 200 ×. Cells with a high versus low/negative TUNEL intensity are indicated by the *black* or *purple arrows*, respectively.

Light microscopic images of the entire tumor area were acquired under 200 × magnification using the Leica Application Suite program, version 4.13.0 (Leica, Wetzlar, Germany). A total of 20 representative fields were selected for each sample except for 7 smaller tumors, which were quantified entirely with a median of 9 fields per tumor (range = 1–16 fields, mean ± standard deviation [SD] = 9.7 ± 5.5). For the quantitative analysis of TUNEL-positive area, color deconvolution of the images was performed by using the Fiji software (Laboratory for Optical and Computational Instrumentation, University of Wisconsin-Madison, Madison, WI, USA, http://imagej.nih.gov/ij; version 1.52p) as described in Ref. 24 to separate the TUNEL signals (blue-green), nuclei (pink), and pigmentation (brown) with minimal overlap. For this purpose, the following user-defined settings for the red, green, and blue components were applied: nuclei (red-1 = 0.482, green-1 = 0.719, and blue-1 = 0.501); TUNEL (red-2 = 0.776, green-2 =: 0.501, and blue-2 = 0.382); pigmentation (red-3 = 0.446, green-3 = 0.616, and blue-3 = 0.649). The TUNEL-positive areas on each image were automatically selected by threshold adjustment (Fig. 1). The percentage of TUNEL-positive area in each tumor was calculated by dividing the sum of the TUNEL-positive areas by the total area that was analyzed. Necrotic areas were included in the quantification of TUNEL-positive DNA breaks due to the necrotic DNA degradation that can occur in early apoptosis^25^ or at the single-cell level,^26^ whereas the vascular regions were excluded from analysis.

### Immunohistochemistry 

Deparaffinized and rehydrated sections were incubated in pre-warmed 10 mM sodium citrate buffer (pH = 6.0) containing 0.025% Polysorbate-20 for 30 minutes in a steam-cooker for antigen retrieval, cooled to room temperature, washed 3 times with PBS for 10 minutes, and incubated in the sterile-filtered blocking buffer (3% bovine serum albumin in 10 mM Tris-HCl [pH = 7.5], 120 mM KCl, 20 mM NaCl, 5 mM ethylenediaminetetraacetic acid, and 0.1% Triton X-100) for 30 minutes at room temperature. For chromogenic immunohistochemistry (IHC), the sections were incubated with the primary antibodies against von Willebrand factor (vWF, rabbit; DAKO, Agilent, Santa Clara, CA, USA; A0082, 1:100 dilution) or CD68 antibodies (rat, Abcam, ab53444, 1:25) overnight at 4°C. The negative control was incubated with the blocking buffer only.

After 3 rinses in PBS, sections were treated with freshly prepared 3% hydrogen peroxide in PBS for 15 minutes, washed twice with PBS, and incubated with HRP-conjugated goat anti-rabbit (Jackson Immunoresearch, Cambridgeshire, United Kingdom, 111-035-003, 1:500 dilution in blocking buffer) or donkey anti-rat secondary antibodies (Jackson Immunoresearch, 712-035-150, 1:500) for 1 hour. For the chromogenic signal detection, the rinsed sections were incubated with freshly prepared HRP Green substrate for 10 minutes and washed in triple distilled water for 5 minutes. After the staining of nuclei with nuclear fast red for 10 minutes, sections were briefly rinsed for 1 minute in triple-distilled water, dehydrated for 30 seconds each in an ascending series of ethanol (75%-96%, 2 × 100%), kept twice for 5 minutes each in xylene, and sealed with Entellan-coated coverslips.

Due to the limited number of paraffin sections from the Tübingen cohort, co-detection of vWF and CD68 was performed by double fluorescent IHC, where the overnight incubation with the abovementioned primary antibodies or mouse anti-CD68 IgG (Santa Cruz Biotechnology, Inc., Heidelberg, Germany, sc17832, 1:50) was followed by a mixture of secondary Alexa 488-conjugated anti-rabbit (Abcam, ab150065, 1:100) and Cy3-conjugated anti-rat (Jackson Immunoresearch, 112-165-072, 1:100) or anti-mouse antibodies (Merck, Darmstadt, Germany, AP124C, 1:100) for 1 hour at room temperature under light protection, omitting the treatment with 3% hydrogen peroxide. The nuclei were counterstained with 0.5 µg/mL 4′,6-diamidino-2-phenylindole in PBS for 10 minutes and the sections were mounted with Mowiol-coated coverslips.

### Combined IHC With Fluorescent In Situ Hybridization (Immuno-FISH)

Fluorescent IHC was performed as described above using the primary antibodies against HMB45 (Abcam, ab787, mouse, 1:100 in blocking buffer) or Melan-A (Abcam, ab210546, rabbit, 1:100 in blocking buffer), followed by the secondary Alexa 488-conjugated anti-mouse antibodies (Abcam, ab150077, 1:100 dilution in blocking buffer) or Cy5-conjugated anti-rabbit antibodies (Jackson Immunoresearch, 111-175-144, 1:50 dilution in blocking buffer) for 1 hour at room temperature under light protection, omitting the treatment with 3% hydrogen peroxide. The sections were then processed for the co-detection of chromosome 3 by the Immuno-FISH assay,^27^ using the centromeric CEP3 Spectrum Orange probe (Abbott, Wiesbaden, Germany). The nuclei were counterstained with 0.5 µg/mL 4′,6-diamidino-2-phenylindole in PBS for 10 minutes and the sections were mounted with Mowiol-coated coverslips.

The sections were analyzed under a fluorescence microscope (Leica DMI 6000B) using the following filter sets: A4 (Excitation = 360/40 nm, Emission = 470/40 nm; nucleus); L5 (Excitation = 460/40 nm, Emission = 527/30 nm; HMB45); Y3 (Excitation = 545/30 nm, Emission = 610/75 nm; chromosome 3); and Y5 (Excitation = 620/60 nm, Emission = 700/75 nm; Melan-A). Images were captured under 400 × magnification using a monochrome digital camera (DFC 350 FX; Leica) connected to the microscope. Image acquisition was performed by using the Leica Application Software (Advanced Fluorescence 2.3.0, build 5131).

### Evaluation of Histopathological Factors

The histopathological factors that were analyzed in this study involved the tumor cell type, mitotic activity, necrosis, fibrosis, vascularization, and macrophage infiltration.^10^^,^^28^^–^^31^ Tumor cell morphology, mitotic figures, as well as the extent of necrosis and fibrosis were evaluated on the paraffin sections of the tumors that were stained with hematoxylin and eosin. Tumors with >90% epithelioid or spindle cells were classified as dominant epithelioid or spindle, respectively, whereas the remaining tumors with >10% epithelioid and <90% spindle cells were categorized as mixed cell morphology.^32^ Mitotic figures were counted in 40 random high-power fields (400 × magnification) excluding the necrotic or heavily pigmented areas. Mitotic figures were identified based on the absence of nuclear membrane, presence of condensed chromosomes, and exclusion of hyperchromatic or apoptotic nuclei.^33^ Due to the small sample size, mitotic figures could not be assessed in 4 of the 15 tumors (27%) that received fSRT. The extent of necrosis and fibrosis was evaluated under 200 × magnification using a light microscope (Leica).

Tumor vascularization was analyzed on the sections that were processed by IHC for vWF.^31^ For this purpose, the light or fluorescent microscopic images of the entire tumor area were acquired under 50 × magnification (Leica). The area of the vWF-positive vessels within the tumor body (excluding the normal choroid) was measured by using the Fiji software and expressed as the percentage of total tumor area. To assess macrophage infiltration, images of the CD68-positive cells were acquired under 200 × magnification using a light or fluorescent microscope (Leica). The sum of the CD68-positive cells was normalized to the total area of the respective tumor and expressed as cells/mm^2^.

### Assessment of the Chromosome 3 Status

The percentage of cells with monosomy 3 and the chromosome 3 index (ratio of the chromosome 3 signals to the number of nuclei) were calculated as described^24^^,^^27^^,^^34^ in the tumor regions that were positive for Melan-A or HMB45. The necrotic domains were excluded due to the very weak chromosome signals. A minimum of 203 cells were evaluated in each sample, except for one small tumor with 88 nonoverlapping nuclei. The positivity for Melan-A or HMB45 was evaluated based on the expression of these proteins in the adjacent retina or choroid in our enucleation samples whereas such a direct comparison with the neighboring regions was not possible in the tumors that underwent endoresection. The chromosome 3 signals in the retina of 3 patients were also quantified as a positive control for diploidy. Due to the absence of a standard cutoff value for defining the presence of monosomy 3 in UM,^35^ the measured parameters were initially sorted based on the median. The median values were calculated separately for the tumors from the Tübingen and Lübeck cohorts owing to the differences in the storage time of these tissue samples, which may influence the quality of the antigen expression or FISH signals.^36^^–^^38^

Both parameters were converted into binary scores based on the median value of the corresponding cohort as follows: percentage of cells with monosomy 3, 0 = <median, 1 = ≥median; chromosome 3 index, 0 = >median, 1 = ≤median. For the final analysis, tumors that scored “1” for both parameters, were designated as “monosomy 3,” whereas the samples that scored “0” by both methods were classified as “disomy 3.” Tumors that scored “1” by only one method were initially defined as “mixed.” For a more even distribution, the mixed tumors with a percentage of >40% or a chromosomal index that was at least one SD below the mean of the entire cohort were considered as “monosomy 3.” The mixed tumors that did not fulfil these criteria were allocated to the “disomy 3” group. The relatively stringent cutoff value of 40% for the percentage of tumor cells was selected based on a previous study that evaluated the thresholds between 5% and 50% for defining monosomy 3 in UM.^35^

### Statistical Analysis

Data were analyzed using the NCSS statistical software (version 22.0.3, Kaysville, UT, USA). The median, mean, minimum, maximum, and SD were calculated as descriptive statistics. The proportions of two categorical variables were examined using the Fisher's Exact test. The association of binary factors with continuous (numerical) values was evaluated by using the Mann-Whitney *U* test with normal approximation and tie correction. The metastasis-free survival rate was evaluated by Kaplan-Meier curves and log-rank test, taking the date of primary tumor diagnosis as the starting point and presenting the 95% confidence intervals (CIs). For the Kaplan-Meier analysis, patients were censored at the end of the follow-up period if they survived without the evidence of systemic metastases or were deceased due to other/indeterminate causes. *P* values less than 0.05 were considered as significant.

## Results

### Patient and Tumor Characteristics

We were interested in the effect that irradiation may have on DNA damage in UM and were able to compare tumors that had been treated with a single dose or multiple (fractionated) doses of stereotactic irradiation with non-treated enucleated UM.

A total of 31 patients (57%) underwent enucleation without previous radiotherapy, whereas the remaining 23 patients (43%) received SRT. One cohort of eight patients (University Eye Clinic Tübingen) was treated with a single (high) dose of SRT (sdSRT), whereas the patients in the second cohort (*n* = 15, University Eye Clinic Lübeck) received fractionated doses (fSRT). The median age of the patients in the combined cohort was 66.5 years, with a range of 37 to 90 years old. A similar age range was found in the different subgroups originating from Tübingen or Lübeck (see Table 1). Further characteristics and statistics regarding gender, tumor size, ciliary body or optic nerve invasion, tumor cell morphology, vascularization, mitotic figures, necrosis, macrophage infiltration, follow-up time, and development of metastatic disease are summarized in Table 1.

Significant differences were found for largest basal diameter (LBD), tumor thickness (TT), and ciliary body invasion. Tumors were smaller and less frequently showed ciliary body involvement (CBI) in the irradiated (SRT) compared to the non-treated samples (LBD: mean ± SD = 14.0 ± 4.4 vs. 16.4 ± 5.6 mm, respectively; *P* = 0.048; TT: mean ± SD = 6.2 ± 2.9 vs. 9.2 ± 4.8 mm, respectively; *P* = 0.017; CBI: 5.6% vs. 30%, *P* = 0.004; see Table 1). Mitotic figures occurred less frequently in the irradiated versus non-irradiated tumors (mean ± SD = 1.6 ± 1.6 vs. 3.1 ± 1.9, respectively, *P* = 0.008; see Table 1). Fibrosis was detected in only one tumor from a non-irradiated, 74-year-old female patient. The extent of fibrosis in this sample was confined to <10% of the tumor area (data not shown).

### Automated Quantification of the TUNEL-Positive Regions in the Primary UM Samples

All the tumors displayed TUNEL-positive cells at various levels. The TUNEL reactivity was mainly confined to the nucleus with minimal cytoplasmic or stromal signals, which may have originated from the truncated nuclei in the paraffin sections. To quantify the area of TUNEL-positive regions, light microscopy images of the specimens were deconvoluted to separate the TUNEL signals from the nuclear staining and pigmentation. With this method, we were able to distinguish the cells with a high versus low/negative TUNEL intensity (black or purple arrows, respectively, see Fig. 1). The TUNEL-positive areas were then automatically selected by threshold adjustment and presented as the percentage of the total area of analysis (see Fig. 1).

### Extent of DNA Damage in the Combined Cohort With Regard to the Radiotherapy and Radiotherapy Regimen, Chromosome 3 Status, and Development of Metastatic Disease

**Figure 2.**
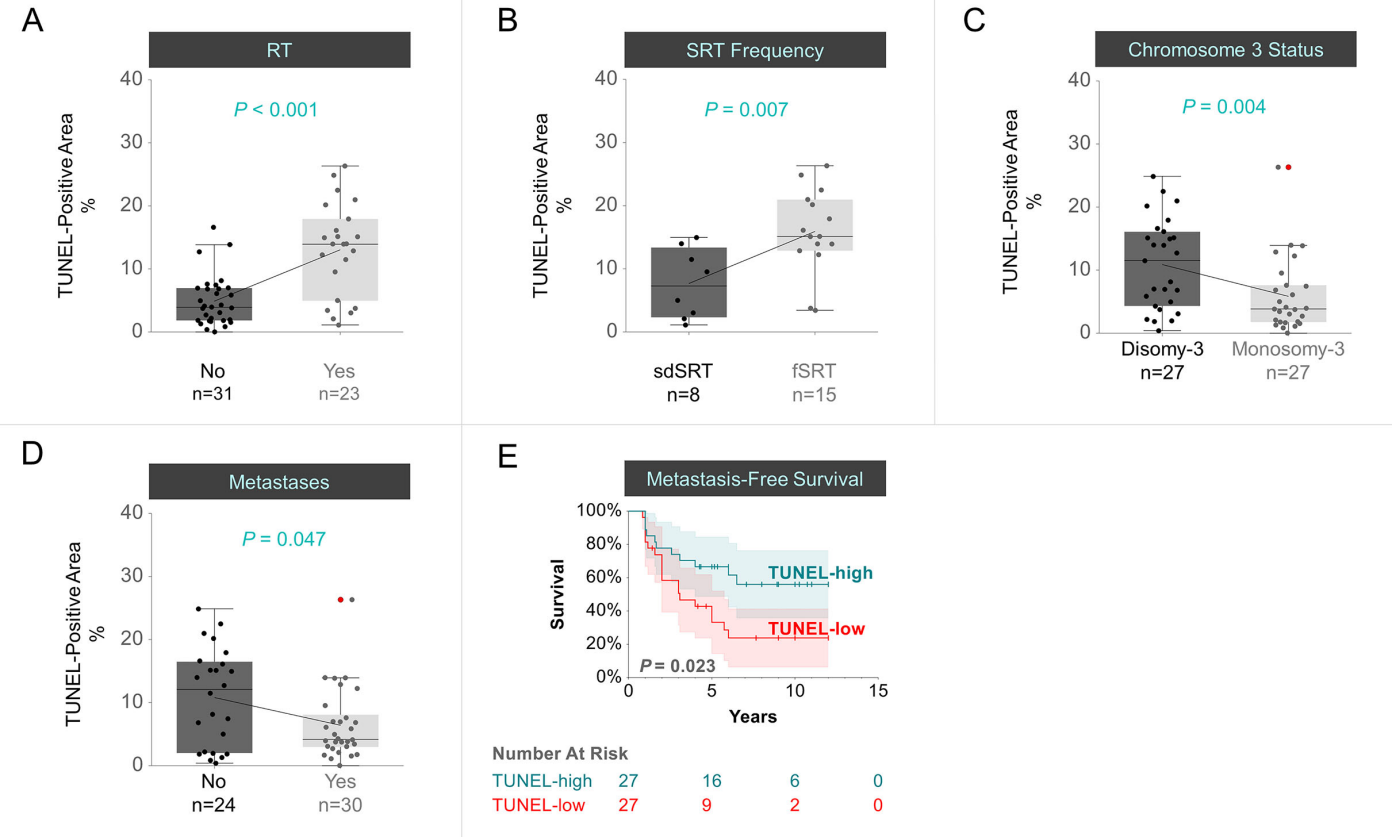
DNA damage in the combined cohort as demonstrated by the TUNEL-positive area. DNA damage was evaluated with regard to the (**A**) radiotherapy (RT) status, (**B**) frequency of stereotactic RT (SRT), (**C**) chromosome 3 copy number, (**D**) development of systemic metastases, and (**E**) metastasis-free survival. In (**A** to **D**), the ratio of the TUNEL-positive area to the total quantified area of each tumor was presented as percentage. The mean values were connected with the *sloped lines*. The *red dots* indicate the severe outliers. The *P* values were evaluated by the Mann-Whitney *U* test. fSRT, fractionated SRT; *n*, number; sdSRT, single-dose SRT. (**E**) Kaplan-Meier estimate of metastasis-free survival with respect to the DNA damage, which was classified based on the median TUNEL intensity (TUNEL-high: >median; TUNEL-low: <median). The x-axis indicates the time elapsed from the diagnosis of the primary tumor until the development of distant metastases or last follow-up. The y-axis demonstrates the percentage of patients that remained metastasis-free. Censored patients are indicated with the vertical ticks whereas the 95% confidence intervals are highlighted as background in the corresponding colors of each group. The number of patients at risk at each time point is presented underneath the curve. The *P* value was assessed by the log rank test.

When looking at all UM samples (*n* = 54), the extent of DNA breaks was 2.7-fold higher (*P* < 0.001) in tumors that had received radiotherapy (*n* = 23; mean ± SD of TUNEL-positive area = 13.1 ± 7.3%) compared to the non-irradiated tumors (*n* = 31; mean ± SD of TUNEL-positive area = 4.9 ± 4.0%; see Table 2; Fig. 2A). Among the irradiated tumors (*n* = 23), a 2.1-fold increase in DNA damage (*P* < 0.007) was detected in response to fSRT (*n* = 15; 15.9 ± 6.6%) compared to sdSRT (*n* = 8; 7.6 ± 5.5%; see Table 2; Fig. 2B). When looking at all tumor samples, monosomy 3 was associated with less DNA damage (*P* = 0.004) by approximately 46% (*n* = 27; mean ± SD = 5.9 ± 5.8%) compared to the disomy 3 tumors (*n* = 27; mean ± SD = 10.9 ± 7.1%; see Table 2; Fig. 2C). Likewise, 41% less DNA breaks (*P* = 0.047) were detected in patients who developed systemic metastases (*n* = 30; mean ± SD = 6.4 ± 5.5%) compared to the patients who remained metastasis-free within the follow-up time (*n* = 24; mean ± SD = 10.8 ± 7.8%; see Table 1, Fig. 2D). Patients with a weaker DNA damage in their primary tumor also had a shorter time until the development of distant metastases compared to the patients having stronger DNA injury, with a metastasis-free survival rate of 33% (95% CI = 14-52%) versus 67% (95% CI = 49-84%), respectively, after 5 years, as estimated by the Kaplan-Meier analysis (*P* = 0.023; log rank test; Fig. 2E).

### Extent of DNA Damage in the Non-Irradiated Versus Irradiated Tumors With Regard to Their Chromosome 3 Status

**Figure 3.**
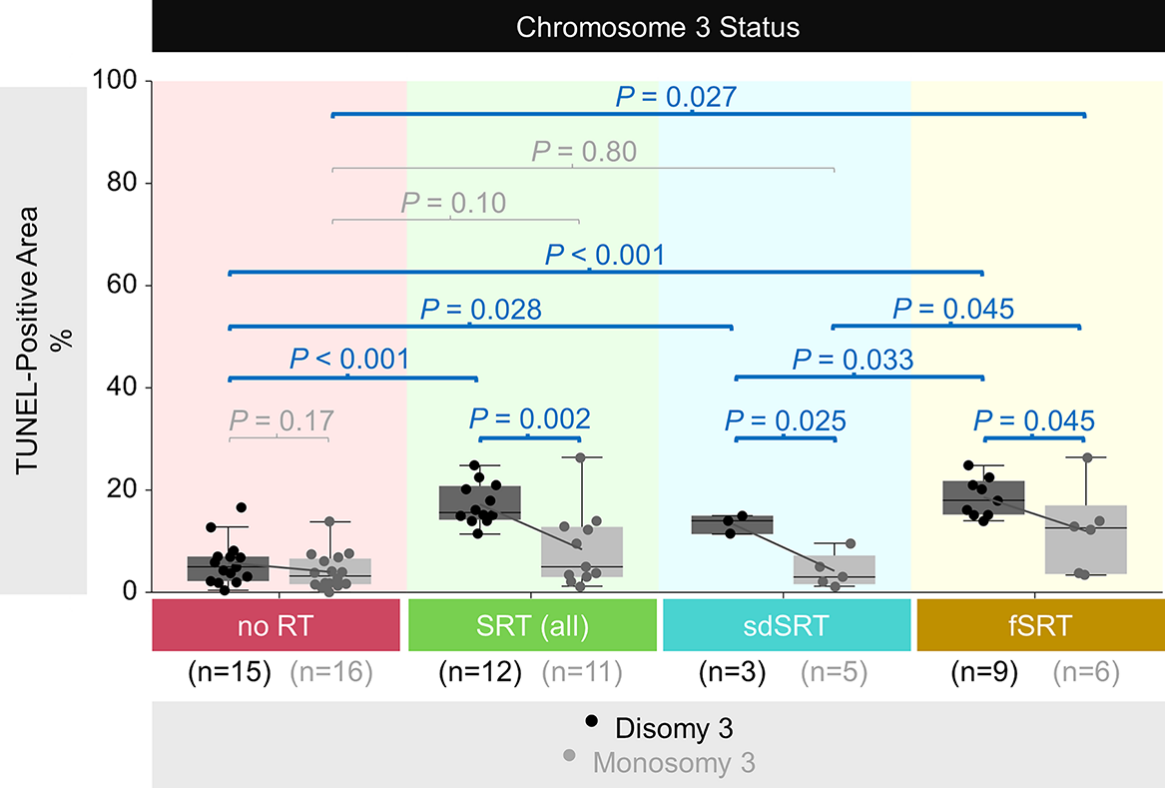
DNA damage with regard to the chromosome 3 status in the tumors that received no radiotherapy (RT) versus stereotactic radiotherapy (SRT). The ratio of the TUNEL-positive area to the total quantified area of each tumor was stated as percentage. The mean values were connected with the sloped lines. The *P* values were determined by the Mann-Whitney *U* test. fSRT, fractionated SRT; *n*, number; sdSRT, single-dose SRT.

When we subdivided the patients with regard to the radiotherapy regimen, we observed less DNA damage in the irradiated tumors with monosomy 3 compared to the disomy 3 tumors both in the combined (SRT, *n* = 23) and 2 individual cohorts (sdSRT [*n* = 8] and fSRT [*n* = 15]; *P* < 0.05; see Table 2; Fig. 3). When looking at the disomy 3 tumors alone, the mean DNA damage was approximately 27% lower in the disomy 3 tumors which had received sdSRT (*n* = 3; mean ± SD = 13.5 ± 1.8%) compared to the disomy 3 tumors with fSRT (*n* = 9, mean ± SD = 18.5 ± 3.8%, *P* = 0.033; see Table 2; Fig. 3). Likewise, monosomy 3 tumors with sdSRT exhibited 65% less mean DNA damage (*n* = 5; mean ± SD = 4.2 ± 3.3%) compared to the monosomy 3 tumors treated with fSRT (*n* = 6; mean ± SD = 12.1 ± 8.4%; *P* = 0.045; see Table 2; Fig. 3).

In contrast, we did not observe a significant difference in the extent of DNA damage in the non-irradiated tumors with monosomy 3 versus disomy 3 (*n* = 15 and 16, respectively; see Fig. 3). Additionally, the non-irradiated samples from the Tübingen and Lübeck cohorts did not exhibit a notable difference in the magnitude of DNA damage either in the disomy 3 or monosomy 3 subgroups (*P* > 0.05; data not shown), weakening the possibility of histological artefacts due to the longer storage time^36^^–^^38^ of the tumors from the former cohort.

### Extent of DNA Damage in the Non-Irradiated Versus Irradiated Tumors With Regard to the Development of Metastases

**Figure 4.**
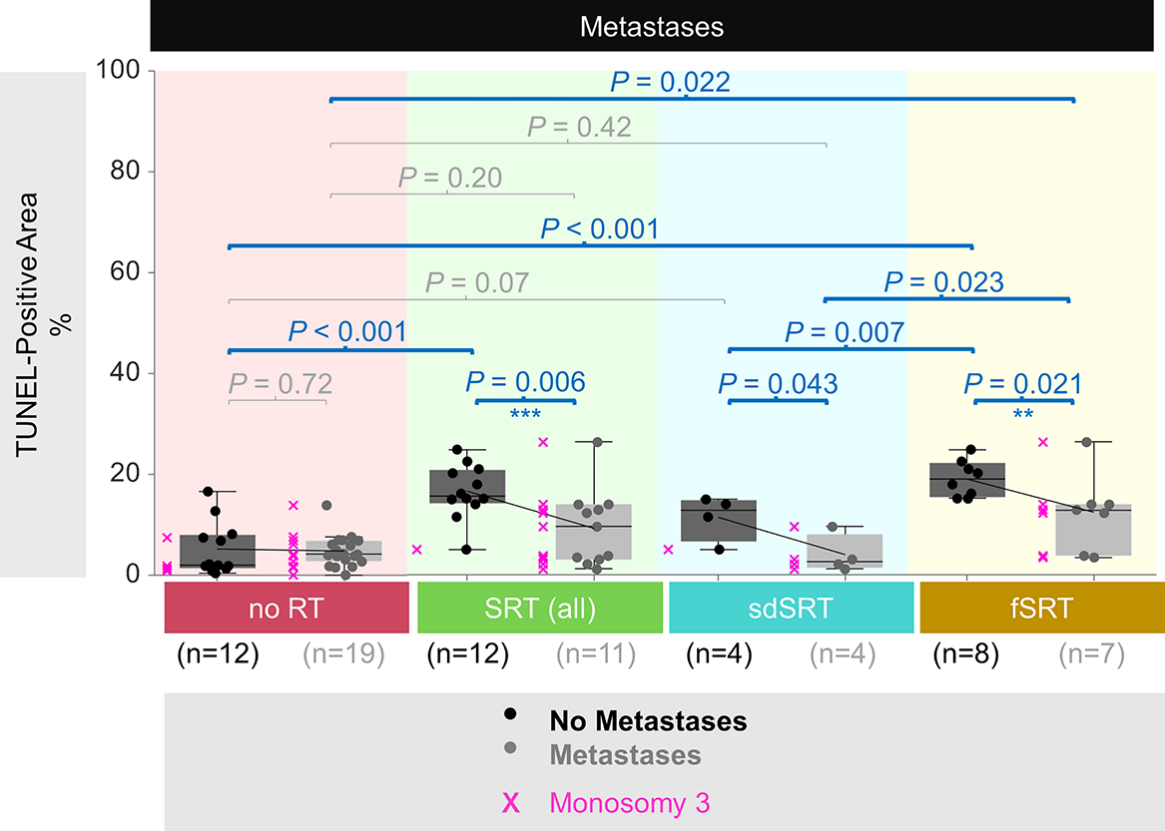
DNA damage with regard to the development of metastases in the tumors that received no radiotherapy (RT) versus stereotactic radiotherapy (SRT). The ratio of the TUNEL-positive area to the total quantified area of each tumor was indicated as percentage. The mean values were connected with the *sloped lines*. The tumors with monosomy 3 were indicated by the *pink* “*x*” symbols. The *P* values were assessed by the Mann-Whitney *U* test for the comparison of TUNEL-positive areas and by Fisher's Exact test for evaluating the ratios of monosomy 3 versus disomy 3 tumors with regard to the development of metastases in each group. **P < 0.005, ***P < 0.001 for the Fisher's Exact test. fSRT, fractionated SRT; *n*, number; sdSRT, single-dose SRT.

In the non-irradiated samples, no significant association was detected between the extent of DNA damage and metastases (Fig. 4). However, the patients who developed metastases despite the radiotherapy exhibited less damage in their primary tumors, particularly after sdSRT compared to fSRT (*P* < 0.05; see Fig. 4). The irradiated patients who developed metastases presented more frequently with monosomy 3 tumors as opposed to the irradiated patients who remained metastases-free among the fSRT group and combined cohort (*P* < 0.005 and *P* < 0.001, respectively; see Fig. 4).

## Discussion

Irradiation of UM enables a high rate of local tumor control, but approximately 10% of the patients develop local recurrence, which increases the risk of fatal metastases.^2^^,^^8^ Although metastatic dissemination may have occurred before therapy, understanding of the factors influencing the local tumor control are relevant. This is, to our knowledge, the first study analyzing the influence of irradiation dose delivery to the tumor together with the monosomy 3 status in the tumor. To elucidate these aspects, we evaluated the amount of DNA breaks with regard to the chromosome 3 status in tumors that received either a single (high) dose (sdSRT) or fractionated (fSRT) stereotactic irradiation with photons or no irradiation at all.

First, our results demonstrate a significant increase in DNA damage in response to SRT. Second, fractionated SRT (fSRT) induces a stronger DNA damage compared to the single (high) dose irradiation (sdSRT). Third, monosomy 3 positive tumors exhibited less DNA breaks in response to radiotherapy compared to the irradiated disomy 3 tumors.

Our findings support fractionated irradiation (fSRT) rather than the single-dose delivery as the more efficient approach to induce DNA damage. This latter contradicts the results of an earlier study^18^ that led us to the single session irradiation in the first cohort of patients in Tübingen. The aforementioned study reported a more profound cell damage in the choroidal melanoma cells that received a high-dose application as opposed to several fractions.^18^ Yet, this study used a two-dimensional culture of UM cells, which mimics the tumor surface that becomes primarily exposed to the irradiation. The exposure of a monolayer of cells to a high dose of ionizing irradiation would indeed be expected to induce a stronger injury compared to a fractionated treatment with lower doses. However, this model may not be adequate for representing a three-dimensional tumor, which retains a source of weakened but viable cells in the deeper layers that can recover and be responsible for recurrences after radiotherapy.^39^

Using the TUNEL assay, we were not able to distinguish the primary DNA damage that is induced by ionizing radiation^40^^–^^42^ from the secondary DNA fragmentation that characterizes the late stages of apoptosis.^43^ We therefore preferred to interpret the TUNEL signals as the extent of DNA damage rather than apoptosis. The TUNEL assay was originally developed for the detection of double-stranded DNA breaks that occur in the later stages of apoptosis.^43^ However, this assay can also label the necrotic cells with extensive DNA fragmentation.^26^ Moreover, necrosis can occur at the single-cell level,^26^ rendering the distinction of necrotic versus apoptotic cells more difficult in the TUNEL assay. We have therefore included the necrotic areas in the TUNEL quantifications. The TUNEL assay could also yield positive signals in nonapoptotic cells that undergo the DNA repair response after sublethal damage with single-stranded DNA breaks.^39^ Because the ionizing radiation can induce both the single- and the more lethal, double-stranded DNA cleavage depending on its dosage and duration,^39^^,^^42^ it would be very interesting to perform further studies on the nature of DNA damage in the irradiated UMs.

In the present study, we also observed a significant increase in DNA damage in the monosomy 3 tumors that received fSRT as opposed to the single-dose (sdSRT) and the non-irradiated samples. However, the irradiated monosomy 3 tumors underwent less DNA injury compared to the irradiated disomy 3 tumors regardless of the dose delivery. This finding adds to the well-known aggressivity of monosomy 3 tumors.

The low radiosensitivity of the monosomy 3 tumors compared to the disomy 3 samples may be based on the differential expression of several genes that are involved in DNA repair. For instance, the expression of *WDR48* and *XPC* is significantly reduced whereas the *PRKDC* gene is upregulated in tumors with monosomy 3.^17^ Both the *WDR48* and *XPC* genes reside on chromosome 3^17^ and both can act as tumor suppressors by promoting apoptosis,^17^^,^^44^^,^^45^ whereas the *PRKDC* gene that supports the growth of breast cancer cells via the activation of p38 MAPK^46^ is located on chromosome 8 (Locus: 8q11.21).^17^ Accordingly, the pharmacological inhibition of PRKDC results in the suppression of proliferation and survival of cultured UM cells.^17^ The 8q amplification is a frequent anomaly that is commonly observed in the monosomy 3 tumors and associated with a worse prognosis.^14^ Such differences in the expression profile of DNA repair proteins may enable the recovery of monosomy 3 cells from sublethal DNA damage or protect these tumors from apoptosis and underlie the lower radiosensitivity of monosomy 3 tumors in our study.

The study could show that the development of metastatic disease in the follow-up correlated inversely with the extent of DNA damage. Patients within the combined SRT group (including both sdSRT and fSRT) with a lesser extent of DNA breaks developed significantly (*P* = 0.006) more frequently metastases in their follow-up (see Fig. 4). This was also seen in the sdSRT (*P* = 0.043) and fSRT (*P* = 0.021) groups. However, for two reasons this association conceivably does not reflect the cause or consequence for the development of metastatic disease. First, tumor cell dissemination occurs years before the primary tumor is treated^27^^,^^47^^,^^48^ and there is therefore no therapy that changes the patient's fate regarding the development of clinically detectable metastases.^2^^–^^4^ Second, the lesser extent of DNA damage correlates with the presence of the well-known risk factor monosomy 3 in these tumors (see Fig. 4).

Our study entails several limitations, such as the restricted sample size, with less than 10 patients in some subgroups and differing duration between irradiation and tissue asservation in the fractionated irradiation cohort. This is based on the retrospective character of the study and individualized approach for every patient in the fSRT group. Surgery with resection of the tumor was only offered when radiation dependent complications of the affected tumor progressed. We also preferred not to obtain tumor biopsies from our irradiated patients due to the risk of ocular complications.^47^ These factors further contributed to the limited sample size of tumors that underwent eye-preserving treatments such as sdSRT or fSRT.

Another limitation may be the duration of sample storage periods especially for the initial cohort treated in Tübingen and we cannot completely exclude the possibility of histological artifacts due to tissue deterioration.^36^^–^^38^ A reconciliating result, however, is the fact that non-irradiated controls from either cohort exhibited similar levels of DNA damage regardless of the chromosome 3 status (data not shown), thereby weakening the possibility of storage-related biases. A further limitation of our study is the absence of tumor biopsies prior to the irradiation procedure, which may have influenced the chromosome 3 evaluations. Ionizing radiation can indeed induce chromosomal anomalies,^40^^,^^41^ which may confound the genotype analyses.

Our findings highlight the superiority of fractionated irradiation compared to single (high) dose radiotherapy (sdSRT). This principle is supported by distinct in vitro studies^11^^,^^12^^,^^39^ and mainly applied in radiotherapy protocols for UM.^19^^,^^49^^,^^50^ Although metastatic dissemination has probably already occurred when UM is treated,^27^^,^^47^^,^^48^ the presence of monosomy 3 is a noteworthy factor that can negatively influence the efficacy of radiotherapy, the mechanisms of which deserve further investigation. With the future perspective of adjuvant systemic therapy for patients with disseminated tumor cells but clinically undetectable metastatic disease, it is imperative to deliver an effective irradiation dose to the primary tumor that would otherwise continue to spread potentially deadly cells.
